# Epizootiological study on spatiotemporal clusters of Schmallenberg virus and Lumpy skin diseases: The case of Russia

**DOI:** 10.14202/vetworld.2018.1229-1236

**Published:** 2018-09-08

**Authors:** Fayssal Bouchemla, Valery Alexandrovich Agoltsov, Sergey Vasilievich Larionov, Olga Mikhailovna Popova, Ekaterina Vladimirovna Shvenk

**Affiliations:** 1 Department of Animal Disease, Veterinarian and Sanitarian Expertise, Faculty of Veterinary Medicine, Vavilov Saratov State Agrarian University, Saratov, Russia; 2 Department of Food Technology, Faculty of Veterinary Medicine, Vavilov Saratov State Agrarian University, Saratov, Russia; 3 Department of Epidemiology and Risk Assessment, Saratov Scientific and Research Veterinary Institute Branch of Federal Research Center on Virology and Microbiology, Saratov, Russia

**Keywords:** geographic area, prevalence, Russia, Schmallenberg and lumpy skin disease

## Abstract

**Aim:**

The submitted article attempts to highlight and specify the development of Schmallenberg virus (SBV) and lumpy skin disease (LSD) in cartographic illustrations, as well as to assess the epizootic situation of these diseases in the world, especially in Russia.

**Materials and Methods:**

Outbreaks (samples were collected from clinically healthy as well as suspected animals in infected areas) were confirmed and reported to the World Organization for Animal Health by veterinary officials representing countries in different geographical regions in the world. The reports showed that ELISA and polymerase chain reaction were used to identify SBV and LSD, taking into account number of infected, dead, and susceptible animals in infection foci since their first registration including in Russia. Once conventional statistical population (arrange data according to the main goal by regions, infected, and dead animals) was defined, a model was installed. A geo-information system, QuickMAP, was used to clarify the disease distribution map, and through the illustrations, analysis values were obtained.

**Results:**

Using information clusters of some epizootological criteria in various territories has demonstrated 1.302 focus of infection of SBV, of which 63.22% were registered in Europe and 36.78% in Russia. The seroprevalence in Russia was about 7.92% of the examined animals. According to the morbidity structure, the causative agent mainly affected cattle (64.76%), small ruminants (33.68%), and goats (1.56%). A global assessment of the effectiveness of primary epizootic diagnosis by practicing veterinarians was 63.19%, i.e., of 100 suspicion reports of SBV, 63.19 cases are confirmed by laboratory methods. A detailed assessment of the types of animals affected by the disease showed that it was easily diagnosed in sheep (70.38%), cattle (60.4%), and goats (48.57%), respectively. In the wild animal species, a significant prevalence was recorded as- 54.5%. In 2016, 1.209 foci of LSD were registered in the world, with 20.548 heads of cattle affected, while 8.5% of them identified in Russia (in 2017, the figure was 7.5%). Different maps had been generated in QuickMAP. Cluster analysis of the infected livestock in different regions in Russia showed that, in 2016, the Chechen Republic, Krasnodar, and Volgograd regions were, respectively, severely, moderately, and mildly affected. In 2017, the situation changed and Saratov, Orenburg regions, and Bashkiria were severely affected. However, the number of outbreaks decreased by 84.81% by contribution to the previous year. Eritrea, Namibia, and South Africa were leading in a cluster of most infected areas in 2017.

**Conclusion:**

Infectious diseases do not know borders. The emergence of SBV and LSD in the territory of the Russian Federation has followed the most common general dynamics of transborder diseases without ignoring details. The epizootic risk from wild animals and favorable climatic conditions is critical to fight against transmission of these diseases in Russia.

## Introduction

In terms of climate warming and trade expansion, the opinion that transmissible diseases are limited only to certain parts, south of the world, is rejected, as they began to emerge and become enzootic in many regions of the world [1]. Diseases such as bluetongue (BT), African swine fever (ASF), Newcastle disease, and avian influenza have begun to get on new atypical areas. There have recently been first outbreaks of diseases in previously uninfected territories of the Russian Federation (RF) as well. These according to the Office International des Epizootics (OIE) and the Rosselkhoznadzor include ASF, BT, other nosocomial infections, Schmallenberg virus (SBV), and Lumpy skin disease (LSD) in the past 10 years (until February 1, 2018) [1,2]. A 2016-2017 epizootiological inspection of different areas in the RF revealed that the dynamics of disease emergence in the country remained critical, with the newly incurred LSD stated as one of the problems in the RF [3].

In 2016 (RF), 1.209 LSD outbreaks had been reported globally, with a total population of 20.548 animals at risk. An incidence of 15% was estimated, and the average lethality rate was 6.9% [4]. SBV as a new disease on the territory of the RF appeared only in the second season after its first appearance in the world (2012), infections are asymptomatic in adult ruminants, with fever, milk drop, and diarrhea, congenital malformations in newborn and abortions [1,2,5]. However, the SBV cases in RF were 36.78% of the total number of cases in the world, indicating the magnitude and seriousness of the disease in the country [1,2]. At first, an exploratory serological examination of 7.92% of animals to the SBV was conducted in RF, to include small ruminants and cattle in the purpose of having an idea if the disease reached their territories and made first suggestions. Wild animals over the world might have been infected, for example, Alpacas, Anatolian water buffalo, Elk, Bison, Red deer, Fallow deer, Roe deer, Sika deer, Yak, Chamois, and Wild boar [6-12]. In a recent study conducted by Rossi *et al*. [13], it was demonstrated that the spectrum of wild animals susceptible to SBV and disease prevalence in these animals was higher than for BT serotype 8 [4]. As a result, current disease surveillance must be focused on wild animal populations to determine their potential as reservoirs of SBV.

These diseases are well illustrated and studied in different regions except Russian. An approach with cartographical illustration relates the occurrence to different potential risk factors and makes a reliable forecast. This attempt is to make clear the development and assessment of SBV and LSD in cartographic illustrations globally and especially, in Russia.

## Materials and Methods

### Ethical approval

The research was an epidemiological study based on reports of tests previously conducted by other workers, and thus, ethical committee approval was not required.

### Study area and information databases

Outbreaks (samples were collected from clinically healthy as well as suspected animals in infected points) were confirmed in different countries all over the world and reported by veterinary officials to the OIE (WAHID interface) in the past 2 years for LSD and SBV. These reports showed that ELISA and polymerase chain reaction were used to identify the LSD and SBV diseases, taking into account a number of susceptible, infected, dead animals, and focus of these infections since their first registration in Russia [1,2].

### Statistical analysis and tests

The model was built on the basic data allowed us to achieve all statistical analyses (level of significance set at 5% [p<0.05]) and had made various maps that simplified our approach. Moreover, through the geoinformation system, QuickMAP (we used the online version) has processed clustering analysis of cases and outbreak incidence (amount of cases/outbreaks by one focus of SBV and LSDs) with mapping.

Once conventional statistical population was defined (an observational study; all focus of infection with the details of infected and dead animals), a model was built. Moreover, in this way, we could know not only the incidence but also assessment and measurement of risk factors. Before such calculations were available, we were obliged to use the methods of hypothesis testing, criterion *χ*^2^.

This program transcripts geostatistical data to maps (have to prepare the data in Excel that should have conformed the given an example in the given QuickMAP instruction, and updated Excel (Microsoft) has such function too). Those defined areas with high-risk forecast and suggest prevention.

Cluster analysis algorithms were used to group a set of objects (areas) in such a way that objects in the same group (degree of risk or infection…).

## Results and Discussion

### SBV epizootic situation evolution in Europe from 2011 to 2017

Since the appearance of SBV in 2011 with its primary cases in Germany and the Netherlands, the virus had demonstrated rapid and wide circulation in Europe. In total, from the 1^st^ day of its appearance until mid-May 2012, there were 3.745 laboratory-confirmed epizootic outbreaks [3,7,14].

At the end of October 2012, SBV was already detected in 14 European countries, and until the end of the season, the disease had affected almost all European member countries [14,15].

The detected outbreaks, which affected more than 8,000 farms in the north of Europe, were confirmed by different laboratory tests. According to the French National Agency for Food Safety, Environment, and Labor (ANSES) [15], the disadvantaged SBV zone was located in Scotland and continental Scandinavian regions such as Norway, Finland, and Sweden. Later, the virus reached new territories of eastern Europe: Estonia, Latvia, Hungary, Slovenia, and Croatia. [2,3,5,7,8,14,15].

Since August 2013, the infection had been tracked in Romania and in other countries in which SBV was already registered in the first wave. The virus in these countries had constantly expanded its borders and had been periodically shown in previously unaffected areas. In France (May 2013), 5,000 outbreaks were registered in small ruminants and cattle. From September 2013 to April 2014, 95 SBV focus of infection was noted, where 81% among the cattle, 17.9% in sheep, and 1% in goats [1,2].

**Figure-1:**
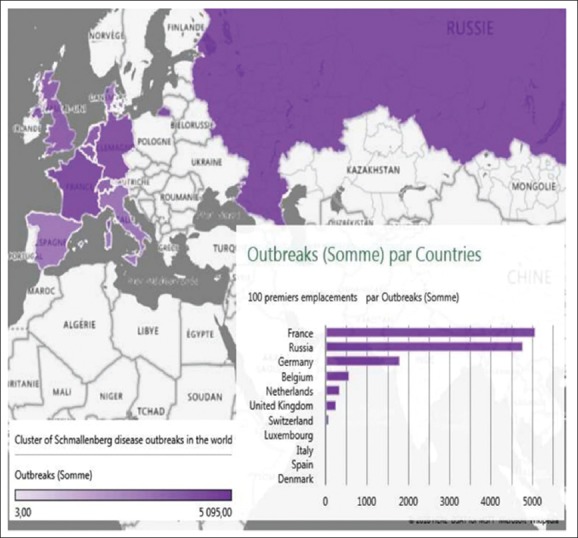
Clustering of Schmallenberg disease outbreaks in the world from 2011 to 2017 [Source: Fayssal Bouchemla].

Taking into account the data published by different specialized agencies on transmissible diseases [1,2,5,6,14,15], we succeeded to make the following picture, describing the recorded outbreaks in certain areas of the world (Figure-1).

In all the 13,021 cases reported in the world, cattle were among the animals affected. The European countries contribution accounted for 63.22% of world outbreaks. SBV was distributed as follows: France - 61.89%, Germany - 22.04%, Belgium - 7.01%, Holland - 4.25%, Great Britain - 3.32%, Switzerland - 1.09%, Luxembourg - 0. 21%, Italy - 0.1%, Spain 0.06%, and Denmark 0.04%. According to the morbidity structure, the causative agent circulated mainly in cattle (64.76% of cases), and among small ruminants, the prevalence was 33.68% (1.56% of them being goats). Analogical research conducted by Berhanu *et al*. [9] under various conditions showed that dairy cattle had a high seroprevalence. Global assessment study of primary diagnosis by practicing veterinarians showed that their reliability was 63.19%, i.e., of 100 reports of clinical (epizootic) suspicion of SBV in various animal species, 63.19 cases were confirmed by laboratory diagnostics. Certainly, we considered it a very effective diagnostics, as the disease had recently appeared.

The analysis of the primary diagnosis by animal species had shown that it is easier for veterinarians to recognize SBV in sheep - 70.38%, cattle - 60.4%, and goats - 48.57%. This indicates that the infection was clearly expressed in sheep than in cattle and goats. Seroprevalence in wild animal species reached 54.54% (average in the world). Attention should be focused on a study carried out by Nick [5] who concluded that the role of wild animals on the phenomena of SBV overwintering should not be overlooked.

### SBV Spatial distribution in the RF

According to the data obtained from *Rosselkhoznadzor*, in RF, 4,789 seropositive cases were registered by February 01, 2018. Of these, 21% were registered during the entire year of 2013 (in I and II quarters - 20% approximately, in III - 10%, and in the last quarter - 50%). The cases were connected with incidences of importation of animals from the infected foreign countries.

To prevent the spread of the disease on the territory of the RF, *Rosselkhoznadzor* issued instruction No FS-AS-7/1154 dated January 1, 2012, which enforced temporary restrictions on the importation of cattle and breeding material from Germany, Netherlands, Belgium, and France [3]. However, in 2016, the virus had been circulating on the territory of the RF but with a low incidence (5.45% seropositivity of the examined livestock [ 20,548 animals]).

**Figure-2:**
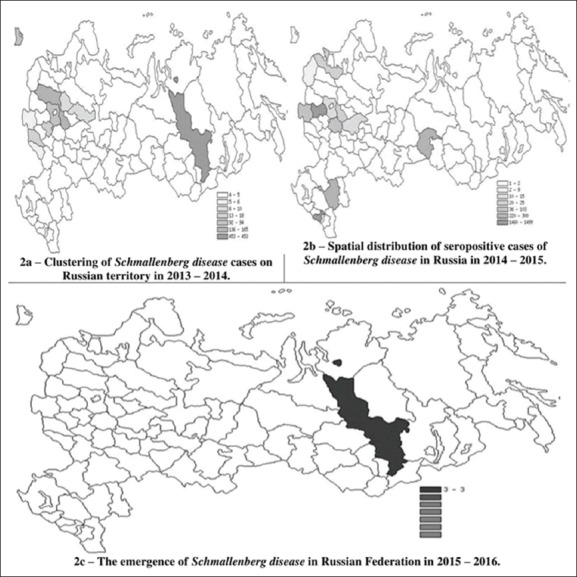
Clustering of Schmallenberg disease prevalence in Russia from 2011 to 2017 [Source: Fayssal Bouchemla].

At the next point of our work, we tried to specify the data on SBV in the RF and graded it to determine the risk factor by areas. In Figure-2, there are maps of the geographical cluster data on SBV in Russia for the period 2013-2017.

Most of the infected cattle with SBV in Russia were imported from infected countries in 2013, (in Krasnodar, Vladimir, and Tver regions, where the risk level was above the average). Areas with average seropositivity (statistical average of positive reaction/all animal [percentage] in different focuses) were located in Kaliningrad and Moscow regions. The lowest level of risk was registered in 2013 in the Republic of Tatarstan, Belgorod and Bryansk regions (Figure-2a).

In 2014-2015, 3,702 blood samples tested seropositive to SBV throughout the RF (the data are presented in Figure-2b - in cattle). During this period, only in the Kaluga region about 40% of SBV cases and, seropositive animals were revealed in the Tyumen and Ryazan regions. Figure-2b shows that the low prevalence of the disease had developed in the Yaroslavl, Tver, Pskov, and Leningrad regions. We noted that, in some of the areas, the SBV previous season had been developing with a high prevalence of morbidity. Positive developments had been achieved because of taking measures to control the pathogen. The prevalence rate decreased, for example, in the Tver region from 13.5% to 0.05%. In 2016 (Figure-2c) in Krasnoyarsk region while the classic enzootic monitoring among cattle, the SBV was discovered with the incidence of 5.45%.

### LSD epizootic situation

Based on the available official data, it is established that the LSD is registered in some African-Asian countries, including the South and Southeast of the Eurasian continent [16,17].

**Figure-3:**
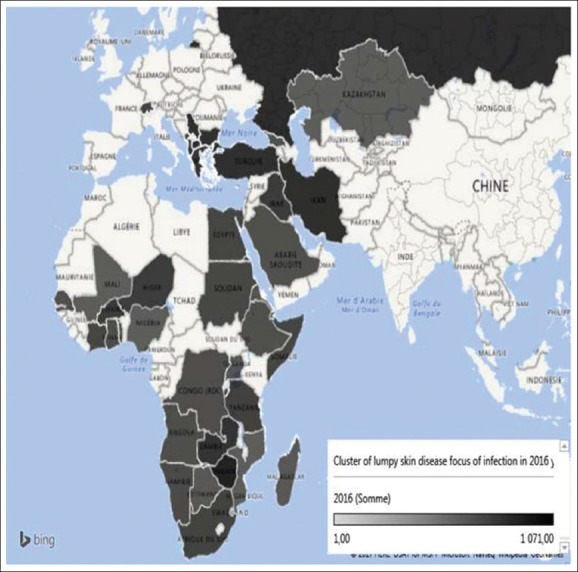
Cluster of Lumpy skin focus of infection in the world in 2016 [Source: Fayssal Bouchemla].

From 2016 to 2017, the LSD amount of outbreaks and endemic countries decreased by 60%, from 3.682 (in 2016) to 891 (2017) (Figure-3a and b) and from 40 to 16 accordingly. In 2017, only 4,463 sick animals were registered over the world [2]. It means that the number of sick animals in 2017 decreased by 93% that was followed by a fall in the overall annual prevalence from 15% (1-23%) to 2% (1-43%) and also with lethality from 7% (0-10%) in 2016 to 2% (0-2%) in 2017.

The distribution of LSD epizootic outbreaks in 2016 shows that 41.82% of all outbreaks occurred in European countries, 42.47% in African, and 15.69% in Asian countries. We must have noted that Zimbabwe was the most affected country (with 30% of all outbreaks). The cartographic analysis of Figure-3 has shown that the worst situation was in Eastern Europe and southern Africa, but in Eastern Africa and the Middle East, the situation was less dangerous. A valued study published in 2017 about the LSD outbreaks in Greece during 2015-16 had cleared the distribution of the disease in the country [18].

**Figure-4:**
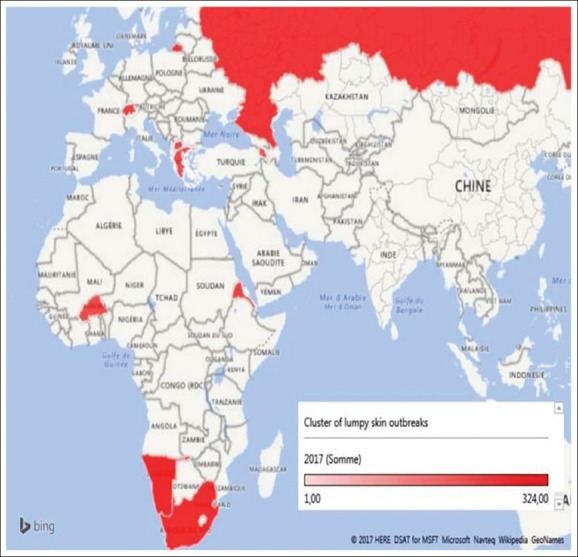
*Lumpy skin* outbreaks cluster in the world in 2017 [Source: Fayssal Bouchemla].

The distribution of outbreaks had changed in 2017: African countries made - 91.23% and European - 8.67%. It should be pointed that, among the nine affected countries in 2017 (Figure-4), only Armenia had not suffered twice from the LSD in the past 2 years, and Namibia had replaced Zimbabwe with the maximum LSD case number (58%). Figure-4 shows that, according to the LSD cluster in 2017, Eritrea, Namibia, and South Africa had a leading position in terms of the number of virus detection (recorded cases+seropositives).

It should be also noted that in Bulgaria in 2016, all livestock that was at risk of disease were killed. The action made it possible for the country to get rid of LSD virus. Therefore, there were no cases of the disease in 2017. Nevertheless, in Macedonia and Greece, where sick animals were killed, the number of outbreaks has reduced but did not prevent the reemergence of infection the following year.

The LSD total incidence average in Europe (in 2016) was 9.24 % (the maximum for countries is 23.3%, in Albania, where in some cases reached 100%, and the minimum - 6.1% was recorded in Greece). The minimum value of the incidence in a particular place was in Greece - 1.3 % [18-20].

The overall mortality rate was 6.11%, except for Greece, where the highest value of this indicator was 18.15%, and in one separately case in this whole study, it reached the value of 77%. The values of mortality were not significant (<1%). In such a study, which was carried out by Birhanu *et al*. [16] an emphasis on risk factors, influencing prevalence in particular areas had been made.

### LSD epizootic situation in Russia

**Figure-5:**
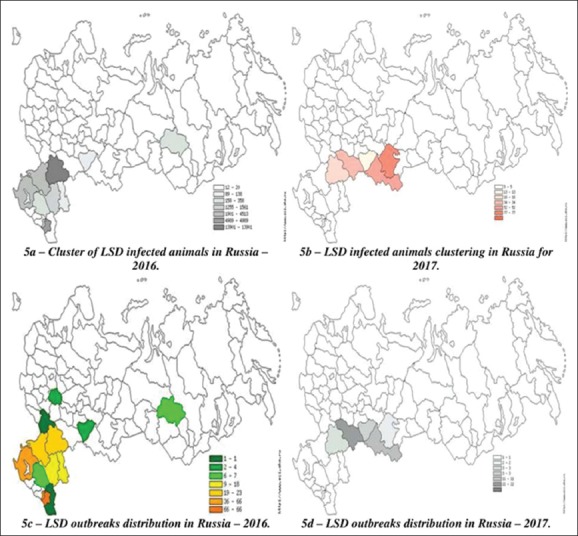
Clustering of Lumpy skin focus of infection and infected animals in Russia in 2016-2017 [Source: Fayssal Bouchemla].

For the first time, LSD focus of infection in Russia arose on May 30, 2016, in Dagestan in 2016 (Figure-5a). The percentage of Russia’s participation according to LSD had not changed much, in 2016, it accounted for 8.5%, and in 2017, it accounted for 7.5%. The available data, from the Rosselkhoznadzor and OIE, show that, at the end of 2016, a large focus of infection of LSD began in the city of Mangyshlak (Kazakhstan), which reached Russia, where prevalence made up 12.9% and lethality about 7.4%. In 2017, a very tense situation had developed on the border of Kazakhstan in Saratov region.

LSD epizootics have caused significant damage to the territory of the RF. Table-1 presents official data on LSD in 2016-2017 in Russia. According to the data in Table-1, we can note a decrease in all characteristics, which indicates the work done by the State Veterinary Service to fight LSD. In 2016, 258 epizootic outbreaks were recorded in the RF, which made up 8.5% of the global foci for the given period in the world. On the territory of the RF, the disease had attacked about 19,000 heads, of which 7% died. In 2017, the situation had improved, as prevalence (up to 2%) and lethality decreased up to 7 times. The epizootic character of this infection in these regions (Figure-5) (the average focus of infection was 73 in 2016 and, in 2017, became 4.69) means that serious effective measures were taken in these zones of Russia, but they were not enough to eradicate the pathogen.

**Table-1:** Summary data on LSD outbreaks and the number of infected and dead cattle during these outbreaks in Russia in 2016-2017.

Years	Outbreaks number	The number of animals in unfavorable areas	Infected animals	Dead animals
2016	258	115.872	18.895	1.332
2017	42	9.254	197	0

The analysis of regional clusters of infected livestock in the RF (Figure-5a) showed that the tensest situation in 2016 developed in the Chechen Republic, Krasnodar Territory, and Volgograd region, where the values of natural LSD focus were above average and reached a value of 170 (the maximum focus of infection in Russia).

The similar situation had emerged in Volgograd region where the prevalence was thrice higher than its average value in Russia. In Samara and Rostov regions, Chechnya and Krasnodar Territory, the registered prevalence was above average too. Moreover, in 2017 in Saratov and Orenburg regions, on the border with Kazakhstan, largest cases took place and the maximum damage caused by LSD, where the prevalence reached 100% in “Dergachi” (Saratov region). Meanwhile, the maximum of the focus of infection dropped to 38.5 and was recorded in Bashkortostan (Figure-5b).

Figure-5c shows that, last year, most of the epizootic outbreaks of LSD were concentrated in Chechnya and Krasnodar Territory (51% of the total infected points) and less in Ingushetia and Rostov region. Single cases were noted in Dagestan and Voronezh region. So far, Bashkiria takes the first place, followed by Saratov and Orenburg regions. In the closed Ulyanovsk region, the epizootic picture of the disease is less intense (Figure-5d).

All this could have been explained by the pathogen circulation from the neighboring countries such as Turkey, Azerbaijan, Iran, and Kazakhstan. Furthermore, the sources of infection including its carriers were found in Dagestan, Chechnya, and other border regions as early as 2015 [1,2]. The seasonal occurrence was quite clear in the RF.

## Conclusion

The epizootic risk from wild animals is a real threat to monitoring as these animals may help to maintain the Schmallenberg pathogen and other reservoirs (wild animals and vectors) of infection. The Southern Federal District of the RF is a zone of the high risk of development of all transboundary infections, by favorable climatic conditions for the causative agent of the disease and its vector, and by geographic location. The spatial and temporal dynamics of LSD is not constant, and its evolution into the Northern regions of Europe is expected, as the ecosystem conditions allow for the existence of the causative virus. These diseases have been followed such dynamics of occurring to Russia like the BTs one.

## Author’s Contributions

FB designed the work. All authors conducted the research work. Data analysis and manuscript were written by FB, VAA, OMP, and EVS under the guidance of FB. All authors read and approved the final manuscript.
